# Molecular mechanism of lysophosphatidic acid-induced hypertensive response

**DOI:** 10.1038/s41598-019-39041-4

**Published:** 2019-02-25

**Authors:** Kuniyuki Kano, Hirotaka Matsumoto, Asuka Inoue, Hiroshi Yukiura, Motomu Kanai, Jerold Chun, Satoshi Ishii, Takao Shimizu, Junken Aoki

**Affiliations:** 1 Laboratory of Molecular and Cellular Biochemistry, Graduate School of Pharmaceutical Sciences, Tohoku University, Sendai, 980-8578 Japan; 2 AMED-CREST, Japan Science and Technology Corporation, Kawaguchi, 332-0012 Japan; 3 AMED-LEAP, Japan Science and Technology Corporation, Kawaguchi, 332-0012 Japan; 4 AMED-PRIME, Japan Science and Technology Corporation, Kawaguchi, 332-0012 Japan; 5 Synthetic Organic Chemistry Laboratory, Graduate School of Pharmaceutical Sciences, University of Tokyo, Bunkyo-ku, Tokyo 113-0033 Japan; 6 Sanford Burnham Prebys Medical Discovery Institute, La Jolla, CA 92037 USA; 7 Department of Immunology, Akita University Graduate School of Medicine, Akita, 010-8543 Japan; 8 Department of Lipid Signaling, National Center for Global Health and Medicine, Shinjuku-ku, Tokyo 162-8655 Japan; 9 Lipidomics, Graduate School of Medicine, University of Tokyo, Bunkyo-ku, Tokyo 113-0033 Japan

## Abstract

Lysophosphatidic acid (LPA) is a blood-derived bioactive lipid with numerous biological activities exerted mainly through six defined G protein-coupled receptors (LPA_1_-LPA_6_). LPA was first identified as a vasoactive compound because it induced transient hypertension when injected intravenously in rodents. Here, we examined the molecular mechanism underlying the LPA-induced hypertensive response. The LPA-induced hypertensive response was significantly attenuated by pretreatment with a Rho kinase inhibitor, which blocks Gα_12/13_ signaling. Consistent with this, the response was weakened in KO mice of LPA_4_, a Gα_12/13_-coupling LPA receptor. KO mice of another Gα_12/13_-coupling LPA receptor, LPA_6_, also showed an attenuated LPA-induced hypertensive response. However, LPA_6_ KO mice also displayed attenuated pressor responses to an adrenergic agent and abnormal blood vessel formation. Using several LPA analogs with varied affinity for each LPA receptor, we found a good correlation between the hypertensive and LPA_4_ agonistic activities. Incubated mouse plasma, which contained abundant LPA, also induced a hypertensive response. Interestingly the response was completely abolished when the plasma was incubated in the presence of an ATX inhibitor. Together, these results indicate that circulating LPA produced by ATX contributes to the elevation of blood pressure through multiple LPA receptors, mainly LPA_4_.

## Introduction

Lysophosphatidic acid (LPA: 1- or 2-acyl-*sn*-glycerol-3-phosphate) is a bioactive lipid that can induce a number of cellular responses, including cell proliferation, migration and cytoskeletal reorganization, most of which are mediated through six defined G-protein-coupled receptors (GPCRs) specific to LPA^1,2^. So far six LPA receptors have been identified, including three that belong to the endothelial differentiation gene (EDG) family (LPA_1–3_) and another three that belong to the P2Y family (LPA_4–6_). LPA is continuously produced in the blood, where both an LPA-producing enzyme, autotaxin (ATX), and its substrate, lysophosphatidylcholine (LPC), are present^3^. Importantly, when plasma is isolated and incubated *in vitro*, a large amount of LPA is produced by the action of ATX^4^.

LPA was originally identified as a vasoactive lipid in soybean extract^5^. Tokumura *et al*. reported that when LPA was intravenously injected in rodents, such as rats and guinea pigs, it induced transient hypertension^6^. In addition, an *i*.*v*. injection of the incubated plasma induced a hypertensive response as was observed for LPA. Human hypertensive patients showed significantly higher levels of LPA in plasma, suggesting that LPA is associated with hypertension^7^. The molecular mechanism underlying the increase of blood pressure by LPA, however, remains largely unknown. LPA-induced hypertension was still observed in LPA_1_ KO, LPA_2_ KO and LPA_1_/LPA_2_ DKO mice^8^, suggesting the involvement of other LPA receptor(s) (LPA_3–6_).

LPA receptor agonists also serve as useful tools to evaluate the role of LPA receptor subtypes. To date, a number of compounds with structures similar to LPA have been developed in several laboratories and were shown to have distinct profiles in activating each LPA receptor^9,10^. We previously designed and synthesized LPA analogs similar to 2-acyl-LPA (LPA with a fatty acid at the *sn*-2 position), so-called “T-series” compounds, in which a ring structure derived from carbohydrates is introduced as a scaffold instead of a glycerol backbone^11^. These analogs have restricted conformational flexibility due to the sugar ring structure and show unique activity for LPA_1–3_ in Ca^2+^ and migration assays. Some compounds, such as T13, were potent ligands for LPA_3_^12^. The reactivity of the T-series compounds to LPA_4_, LPA_5_ and LPA_6_ have not been examined so far.

To clarify the molecular mechanism underlying the LPA-induced pressor response, we examined whether the LPA receptors cloned so far (LPA_1–6_) are involved in LPA-induced transient hypertension by utilizing a combination of LPA receptor KO mice and LPA analogs. Here we report that LPA_4_ is the major hypertensive LPA receptor. Furthermore, we demonstrate that LPA_6_ signaling is crucial for normal vasoactivity and vascular development.

## Results

### LPA administration induces transient hypertension in mice

**Figure 1:**
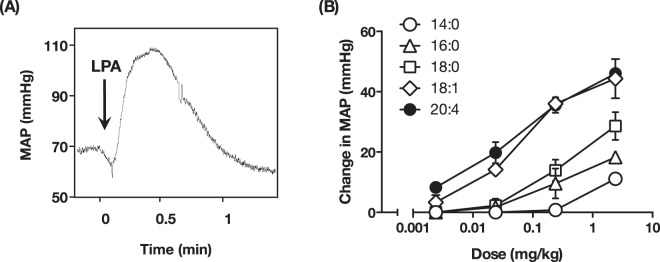
LPA induces transient hypertension in mice. (**A**) Original recording of mice blood pressure. LPA (1-oleoyl, 1.4 mg/kg) was intravenously injected into anesthetized mice to monitor change in blood pressure. Arrow indicates the time point of injection of LPA. (**B**) The increase of mean artery pressure (MAP) in mice injected with the indicated dose of five LPA species was analyzed. Unsaturated LPA (18:1- and 20:4-LPA) showed potent hypertensive activity. Data represents change in MAP as mean ± S.E. (n = 4).

As was demonstrated in other experimental animals, administration of LPA (18:1-LPA, 1.4 mg/kg, *i*.*v*.) in ICR mice produced a weak hypotension followed by a transient hypertension that lasted for a minute (Fig. 1A). This LPA-induced hypertension was observed regardless of mouse strain and anesthetic (data not shown). The LPA-induced hypertensive response was dose-dependent and was observed at concentrations as low as 0.014 mg/kg for oleoyl (18:1)-LPA (Fig. 1B). The hypertensive activity of LPA was dependent on the acyl chain of LPA. Among the five LPA species tested, 18:1- LPA was the most potent in elevating blood pressure and myristoyl (14:0)-LPA was the least potent.

### LPA induces a transient hypertension via the Gα_12/13_-Rho/ROCK pathway

**Figure 2:**
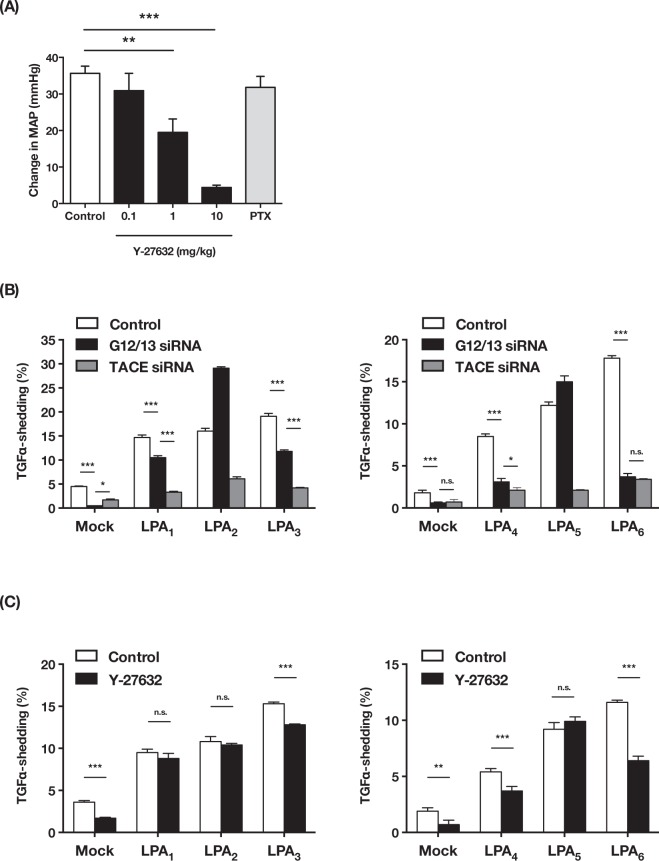
Putative hypertensive LPA receptor(s) couple with Gα_12/13_ protein. (**A**) Effects of GPCR signaling inhibitor to LPA-induced hypertension. Y-27632 significantly inhibited LPA-induced hypertension. Increase in MAP represent as mean + S.E. (n = 7 Control group, n = 3 Y27632 and PTX group **P < 0.01, ***P < 0.001). (**B**,**C**) AP-TGFα release responses of LPA receptors (LPA1-6). LPA1-6 expressing HEK293 cells were stimulated with 18:1-LPA (3 μM). (**B**) Cells were transfected with control siRNA, Gα_12/13_ siRNA or TACE siRNA. (**C**) Cells were pretreated with Y27632 (10 μM). Data represents as mean + S.D. (n = 3, *P < 0.05, **P < 0.01, ***P < 0.001, n.s.: not significant).

To reveal the intracellular signals underlying LPA-produced hypertension, we tested two GPCR signaling inhibitors (Y-27632 and PTX). We found that Y-27632, an inhibitor of a Rho kinase that is activated downstream of Gα_12/13_-Rho signaling, significantly and dose-dependently attenuated the hypertensive activity of LPA (Fig. 2A). In contrast, PTX, which inactivates Gα_i/o_ proteins, had no effect. We observed that bradycardia evoked by acetylcholine *i*.*v*. administration was attenuated by PTX pretreatment (data not shown), confirming that Gα_i/o_ proteins were inactivated. These results suggest that LPA induces a transient hypertension via putative LPA receptor(s) coupling with Gα_12/13_ protein.

Unlike other GPCR assay methods, the TGFα-shedding assay, a novel assay system for detecting GPCR activation that we developed recently^13^, effectively detects Gα_12/13_ signaling as well as Gα_q_ signaling. Importantly, activation of all six LPA receptors was detectable using this assay. To evaluate possible coupling of each LPA receptor to Gα_12/13_ protein, we knocked down both Gα_12_ and Gα_13_ by siRNA. Simultaneous knockdown of Gα_12_ and Gα_13_ dramatically decreased the activity of LPA_4_ and LPA_6_, as was observed for knockdown of TNFα-converting enzyme (TACE) (Fig. 2B), a crucial enzyme in the TGFα-shedding assay (Fig. 2B). In agreement with this observation, Y-27632 significantly reduced the activities of LPA_3_, LPA_4_ and LPA_6_ (Fig. 2C). In LPA_3_-expressing cells, Gα_12/13_ knockdown partially inhibited the TGFα-shedding responses. These data indicate that LPA_4_ and LPA_6_ are the LPA receptors that mainly coupled with Gα_12/13_ protein.

### Attenuated LPA-induced hypertensive response in both LPA_4_- and LPA_6_-deficient mice

**Figure 3:**
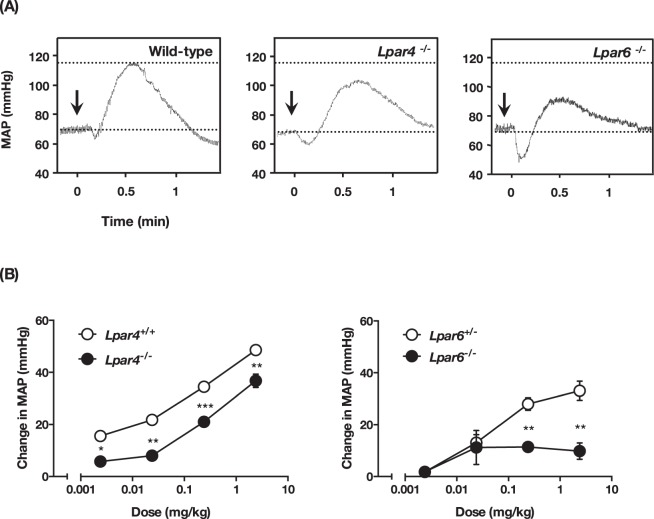
LPA-induced hypertension is attenuated in LPA_4_- and LPA_6_-deficient mice. (**A**) Original recording of mice blood pressure. LPA (1.4 mg/kg) was intravenously injected into mice. Arrow indicates the time point of injection of LPA. (**B**) The increase of MAP in KO mice and their control littermates. Mice were injected with the indicate dose of LPA. Data represents change in MAP as mean ± S.E. (n = 4 *Lpar4*^+/+^, n = 3 *Lpar4*^−/−^, *Lpar6*^+/−^ and *Lpar6*^−/−^ mice, *P < 0.05, **P < 0.01, ***P < 0.001).

To identify the LPA receptors involved in the pressor effect, we tested five single LPA receptor KO mice (*Lpar1*, *Lpar2*, *Lpar3*, *Lpar4* and *Lpar6* null). Consistent with a previous report^8^, administration of LPA in *Lpar1* and *Lpar2* null mice induced a similar hypertensive response as was observed in wild-type mice (Fig. S1). Similar results were obtained with *Lpar1*/*Lpar2* double KO mice (data not shown). The LPA-evoked pressor response was also not affected in LPA_3_-deficient mice, although the hypotensive response was attenuated (Supplementary Fig. 1). These data suggest that *Edg*-type LPA receptors (*i*.*e*. LPA_1_, LPA_2_ and LPA_3_) are not involved in the LPA-induced hypertensive response, at least in mice. We next tested LPA_4_ and LPA_6_ KO mice and found that they showed an impaired pressor response to LPA (Fig. 3A). The dose-response curve of LPA-induced hypertension is shown in Fig. 3B. LPA_4_-deficient mice showed a slightly but significantly lowered response to various LPA dosages. In LPA_6_-deficient mice a lower dosage of LPA (0.014 mg/kg) induced hypertensive responses similar to those in wild-type mice. However, the responses induced by higher LPA dosages were significantly lower in LPA_6_-deficient mice. To address the redundant role of LPA_4_ and LPA_6_ in the pressor effect of LPA, we tried to generate mice lacking both *Lpar4* and *Lpar6* (*Lpar4*/*Lpar6*-double null mice). However, *Lpar4*/*Lpar6*-double null pups produced by intercrossing *Lpar4*^+/−^ *Lpar6*^+/−^ female and *Lpar4*^+/Y^ *Lpar6*^+/−^ male mice were not born (Table 1). Although *Lpar4*^+/−^ *Lpar6*^−/−^ and *Lpar4*^−/Y^ *Lpar6*^+/−^ mice, which retained only one wild-type allele, were born fertile, the number of offspring was less than the value expected from the Mendelian ratios. Further mating experiment using *Lpar4*^−/−^ *Lpar6*^*+/−*^ female and *Lpar4*^−/Y^ *Lpar6*^+/−^ male also failed to generate *Lpar4*/*Lpar6*-double null mice, suggesting that complete loss of *Lpar4*/*6* results in embryonic lethality or death after parturition, while a single remaining wild-type allele is sufficient for normal development and reproduction. We thus could not test *Lpar4*/*Lpar6*-double null mice for LPA-induced transient hypertension.

**Table 1:** Number of offspring of each genotype.

	Offspring Genotype (*Lpar4*)/(*Lpar6*)												Total
	Number of offspring (percentage of total)												
Parental genotype (*Lpar4*)/(*Lpar6*)	Female						Male						
Female × Male	(+/+)/(+/+)	(+/+)/(+/−)	(+/+)/(−/−)	(+/−)/(+/+)	(+/−)/(+/−)	(+/−)/(−/−)	(+/Y)/(+/+)	(+/Y)/(+/−)	(+/Y)/(−/−)	(−/Y)/(+/+)	(−/Y)/(+/−)	(−/Y)/(−/−)	
(+/−)/(+/−) × (+/**Y**)/(+/−)	**15 (11.7)**	**20 (15.6)**	**6 (4.7)**	**14 (10.9)**	**18 (14.1)**	**3 (2.3)**	**13 (10.2)**	**21 (16.4)**	**5 (3.9)**	**6 (4.7)**	**7 (5.5)**	**0 (0)**	**129**
	**Offspring Genotype (** ***Lpar4*** **)/(** ***Lpar6*** **)**												**Total**
	**Number of offspring (percentage of total)**												
**Parental genotype (*Lpar4*)/(*Lpar6***)	**Female**						**Male**						
**Female × Male**	**(+/−)/(+/+)**	**(+/−)/(+/−)**	**(+/−)/(−/−)**	**(−/−)/(+/+)**	**(−/−)/(+/−)**	**(−/−)/(−/−)**	**(+/Y)/(+/+)**	**(+/Y)/(+/−)**	**(+/Y)/(−/−)**	**(−/Y)/(+/+)**	**(−/Y)/(+/−)**	**(−/Y)/(−/−)**	
(+/−)/(+/−) × (−/**Y**)/(+/−)	**13 (10.7)**	**27 (22.3)**	**4 (3.3)**	**9 (7.4)**	**6 (5.0)**	**0 (0)**	**11 (9.1)**	**21 (17.4)**	**11 (9.1)**	**8 (6.6)**	**11 (9.1)**	**0 (0)**	**120**
	**Offspring Genotype (** ***Lpar4*** **)/(** ***Lpar6*** **)**												**Total**
	**Number of offspring (percentage of total)**												
**Parental genotype (*Lpar4*)/(*Lpar6***)	**Female**						**Male**						
**Female × Male**	**(+/−)/(+/+)**		**(+/−)/(+/−)**		**(+/−)/(−/−)**		**(−/Y)/(+/+)**		**(−/Y)/(+/−)**		**(−/Y)/(−/−)**		
(−/−)/(+/−) × (−/**Y**)/(+/−)	**6 (20.7)**		**0 (0)**		**8 (27.6)**		**6 (20.7)**		**9 (27.6)**		**0 (0)**		**29**

### Abnormal vasoactivity and vascular development in LPA_6_-deficient mice

**Figure 4:**
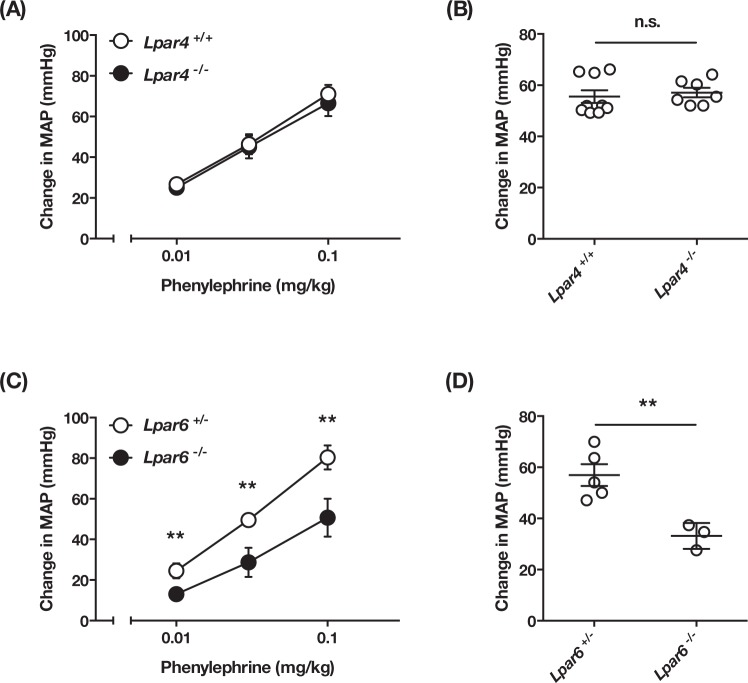
Vasoactive response is impaired in LPA_6_-deficient mice. (**A**,**C**) The increase of MAP in LPA_4_-deficient (**A**) and LPA_6_-deficient mice (**C**) injected with the indicated dose of phenylephrine. Data represents change in MAP as mean ± S.E. (n = 5 *Lpar4*^+/+^, n = 3 *Lpar4*^−/−^, n = 4 *Lpar6*^+/−^ and *Lpar6*^−/−^ mice, **P < 0.01) (**B**,**D**) The increase of MAP in LPA_4_-deficient (**B**) and LPA_6_-deficient mice (**D**) injected with norepinephrine (0.03 mg/kg). Data represents change in MAP as mean ± S.E. (**P < 0.01, n.s.: not significant).

**Figure 5:**
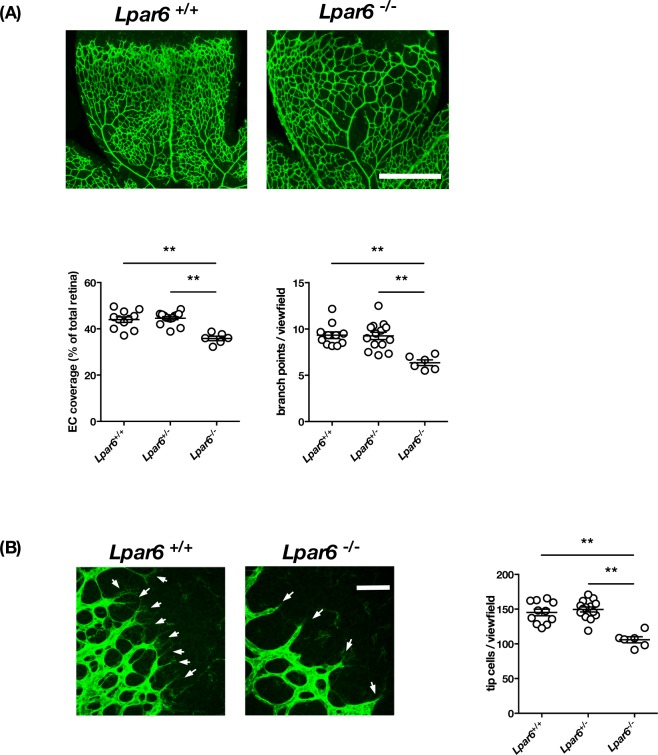
LPA_6_-deficient mice show abnormal vascular structure in retina. (**A**) Isolectin-B4 angiography in wild-type and LPA_6_-deficient mice at P6. *Lpar6*^−/−^ mice displayed a reduction in the density of the vascular network correlating with a decreased number of branches. Data are shown as mean ± S.E. (**P < 0.01) (**B**) Magnification view of angiogenic front in retina. Arrow indicates the tip cell. The number of tip cells were decreased in *Lpar6*^−/−^ mice. Data are shown as mean ± S.E. (**P < 0.01) Scale bars: 500 μm (A); 50 μm (B).

To examine whether the attenuated hypertensive response induced by LPA in LPA_4_ and LPA_6_-deficient mice results from their abnormal vasculature, we next examined the vasoactivity of these mice. We confirmed that both LPA_4_- and LPA_6_-deficient mice had normal blood pressure and heart rate under normal conditions (Supplementary Fig. 2). LPA_4_-deficient mice showed hypertensive responses to phenylephrine similar to those observed in wild-type mice (Fig. 4A). LPA_4_-deficient mice also showed normal pressor responses to norepinephrine (Fig. 4B), indicating that their vasoactive response is not affected in LPA_4_-deficient mice despite the presence of some abnormalities in the blood vascular system of neonates as previously reported^14^. By contrast, LPA_6_-deficient mice displayed significantly lowered responses to both phenylephrine and norepinephrine (Fig. 4C and D), raising the possibility that they have abnormal vasculature that results in impaired vasoactivities. We thus analyzed postnatal retinal blood vessel formation in LPA_6_-deficient mice, a widely used evaluation system for physiological angiogenesis. Isolectin B4 staining, which visualizes the vascular network in the retina, revealed a decreased vascular density and branching in LPA_6_-deficient mice (Fig. 5A). In addition, LPA_6_-deficient vessels extended few filopodia at the vascular front where most endothelial tip cells are located (Fig. 5B). Quantitative analyses of the retinal vessels showed 22% less EC coverage, 26% less branching points and 35% fewer tip cells number in LPA_6_-deficient mice (Fig. 5A,B). Accordingly, an LPA_6_ signal was found to be essential for normal vasculature. Thus, we could not conclude that LPA_6_ is involved in LPA-induced hypertension using LPA_6_-deficient mice, even though they had weakened LPA-induced pressor responses (Fig. 3B).

### Hypertensive activity of LPA analogs, T-series compounds

**Figure 6:**
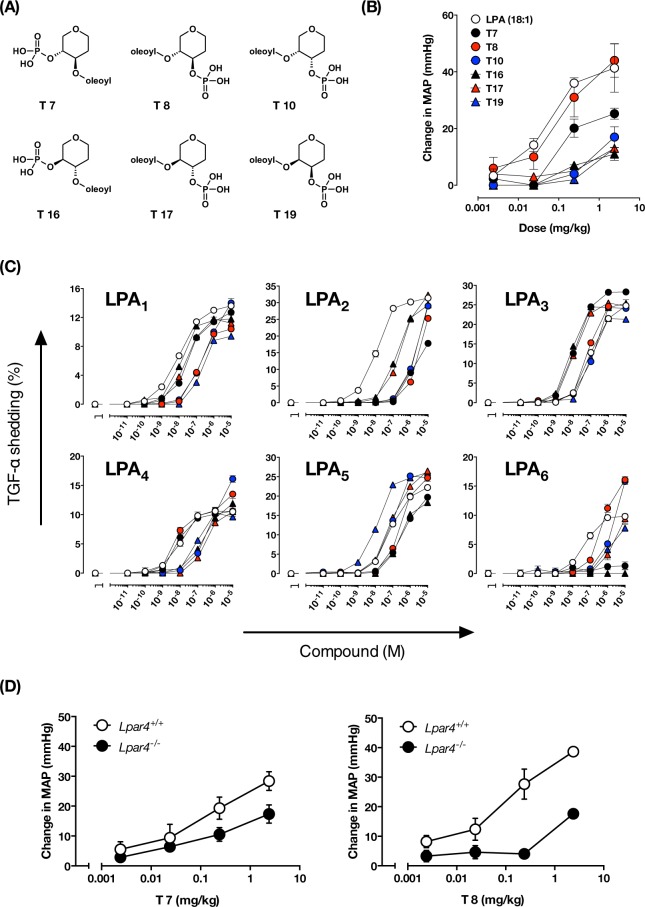
The structure-activity relationship of T-series compounds as a vasopressor and as an LPA receptor agonist. (**A**) Structures of T-series synthesized based on 2-Oleoyl LPA as the lead compound. (**B**) Hypertensive activity of the T-series compounds. The indicated dose of LPA and T-series compound were intravenously injected into ICR mice, and the potencies of these compounds to induce hypertension was analyzed. Data represented as mean ± S.E. (n = 3) (**C**) Agonist activity of T-series compounds against the six LPA receptors. HEK293 cells expressing LPA_1–6_ were stimulated with the indicated concentration of LPA and T-series. Data represented as mean ± S.E. (n = 3) (**D**) MAP increases in LPA_4_-deficient mice injected with the indicated dose of T7 and T8. Data represents change in MAP as mean ± S.E. (n = 3).

To determine the LPA receptors involved in LPA-induced hypertension, we next used a pharmacological approach using LPA analogs called T-series compounds (Fig. 6A). The T-series compounds were designed to identify specific active conformations of the glycerol backbone of LPA by using carbohydrates with a fixed ring structure as scaffolds^11^. We tested the hypertensive activity of six T-series compounds in mice and found that T8 was the most potent in increasing blood pressure (Fig. 6B), with an activity almost equal to that of LPA (18:1). T7, an LPA analog with an acyl-chain and phosphate in the opposite position to T8, also showed a pressor effect but it was less potent than T8 and LPA. In contrast, T10, T17 and T19, which are stereoisomers of T8, and T16, which is a stereoisomer of T7, were much less potent. These data indicate that putative LPA receptor(s) involved in the LPA-induced hypertensive response strictly recognize the structure of these LPA analogs.

### The best vasopressor, T8, is a potent ligand for LPA_4_

Next, we evaluated the ability of the T-series compounds to activate the six LPA receptors (Fig. 6C, Table 2). For LPA_1_, LPA_2_ and LPA_6_, LPA was found to be the best ligand, while some T-series compounds were the best in activating LPA_3_, LPA_4_ and LPA_5_. The weaker vasopressor compounds (T16, T17 and T19) were found to be potent in activating LPA_1_, LPA_3_ and LPA_5_ (EC_50_ < 100 nM). For example, T16 and T17 were potent agonists for both LPA_1_ and LPA_3_, and T19 was a potent agonist for LPA_5_, indicating that LPA_1_, LPA_3_ and LPA_5_ are not the LPA receptors involved in the LPA-induced hypertensive response. Interestingly, T8, the most potent vasopressor compound, was found to be a potent ligand for both LPA_4_ and LPA_6_. The second most potent pressor substance, T7, activated LPA_4_, but not LPA_6_.

**Table 2:** Estimates of EC50, Emax and RIA in TGF-α shedding assay.

		LPA (18:1)	T7	T8	T10	T16	T17	T19
LPA_1_	Emax (%)	13.7	12.5	10.5	15.4	11.7	11.6	9.5
	EC50 (nM)	10	35	130	390	12	21	170
	RIA	1	0.26	0.062	0.030	0.71	0.41	0.043
LPA_2_	Emax (%)	31.2	18.5	33.9	34.3	29.7	33.6	36.1
	EC50 (nM)	11	1000	3800	2200	160	310	2500
	RIA	1	0.006	0.003	0.005	0.066	0.038	0.005
LPA_3_	Emax (%)	25.1	28.5	25.7	24.5	25.1	25	21.8
	EC50 (nM)	97	13	73	120	7.6	11	93
	RIA	1	8.5	1.4	0.77	13	8.9	0.90
LPA_4_	Emax (%)	10.6	10.4	12.3	18.2	12.5	10.8	10.1
	EC50 (nM)	11	7.5	8.2	730	250	270	84
	RIA	1	1.4	1.5	0.025	0.050	0.040	0.12
LPA_5_	Emax (%)	22.2	21.2	25.3	25.5	18.7	26.7	25.7
	EC50 (nM)	72	390	270	85	240	87	12
	RIA	1	0.18	0.30	0.98	0.26	0.99	6.7
LPA_6_	Emax (%)	10	ND	16.8	22.6	ND	ND	7.8
	EC50 (nM)	47	>1000	520	4000	>1000	>1000	970
	RIA	1	ND	0.15	0.027	ND	ND	0.038

ND: not determined.

We then injected LPA_4_-preferred compounds (T7 and T8) into LPA_4_-deficient mice. Hypertensive activity of T8 dramatically disappeared in LPA_4_-deficient mice (Fig. 6D), showing the involvement of LPA_4_ in T8-induced hypertension. Because T8 was a potent ligand for LPA_6_ (Fig. 6C) and LPA_6_ is intact in LPA_4_-deficient mice, it is reasonable to assume that LPA_6_ is not the receptor involved in LPA-induced hypertension. By contrast, hypertensive activity of T7 was significantly attenuated in LPA_4_ KO mice, confirming again the involvement of LPA_4_ (Fig. 6D). However, the T7–induced hypertension still remained in LPA_4_ KO mice, raising the possibility that LPA target(s) other than LPA_4_ are involved. Such targets do not include LPA_6_ because T7 was found to be a poor agonist for LPA_6_ (Fig. 6C).

### ATX-producing LPA in incubated mouse plasma induces transient hypertension

**Figure 7:**
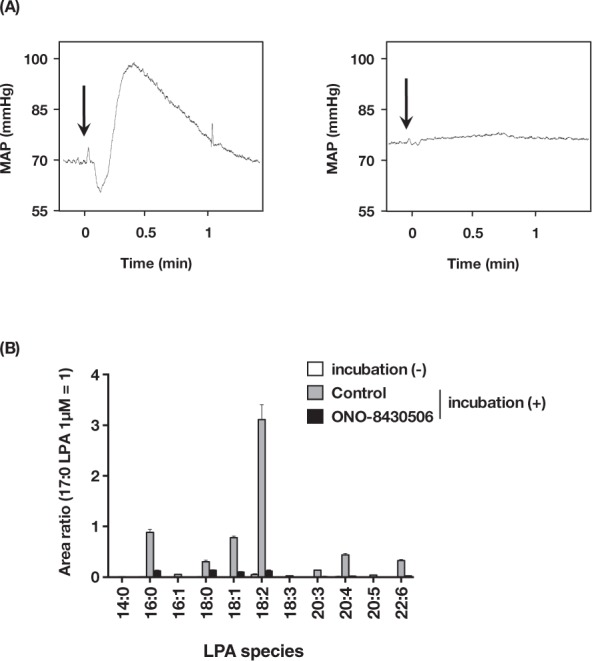
ATX-producing LPA in incubated mouse plasma induces transient hypertension. (**A**) Change in blood pressure after administration of incubated mouse plasma. Each incubated plasma (left: control, right: containing ONO-8430506) was intravenously injected into mice. Arrow indicates the time point of injection. (**B**) LPA levels in incubated mouse plasma. Plasma samples containing vehicle or ONO-8430506 (10 μM) were incubated at 37 °C for 3 hr. Data represents relative abundance, which is the ratio between analyte and internal standard (17:0 LPA, 1 μM) peak area, as mean + S.E. (n = 3).

Incubated plasma is known to contain vasoactive compounds in rats and guinea pigs^15^. LPA has been assumed to be an active component of the pressor response because abundant LPA is accumulated in incubated plasma by the action of ATX. In fact, injecting mice with incubated mouse plasma induced transient hypertension (Fig. 7A), as was observed with LPA (Fig. 1A). After 3 hr incubation, the level of plasma LPA was markedly increased to about ~10 µM, which corresponds to the LPA dosage (0.014 mg/kg) in Fig. 1B. This LPA level was significantly lowered by the ATX inhibitor (ONO-8430506) treatment (Fig. 7B). Interestingly, the hypertensive response was not induced by incubated mouse plasma prepared in the presence of the ATX inhibitor (Fig. 7A). These results clearly showed that LPA produced in the incubated plasma by ATX is responsible for the hypertensive activity of the plasma.

## Discussion

In this study we examined the mechanism underlying the pressor activity of LPA using several tools, including an inhibitor of an LPA-producing enzyme, five LPA receptor deficient mice and LPA analogs with different affinities for each LPA receptor. First, when mouse plasma was incubated with an ATX inhibitor, we were unable to detect the hypertensive activity of the plasma. Production of LPA in the plasma was completely suppressed by the inhibitor, showing clearly that LPA is the hypertensive substance in the incubated plasma.

Among the five LPA receptor deficient mice (LPA_1–4_, and LPA_6_), LPA_4_ and LPA_6_-deficient mice had a partially but significantly attenuated LPA-induced hypertensive response (Fig. 3). Comparison between the hypertensive and the LPA receptor agonistic activities of T-series compounds suggested that LPA_4_ is the most probable hypertensive LPA receptor (Fig. 6 and Table 2). Notably, the hypertensive activity of the most potent vasopressor compound T8 was almost completely suppressed in LPA_4_-deficient mice. Although we did not test LPA_5_-deficient mice, the involvement of LPA_5_ could be excluded since one of the T-series compounds, T19, which was found to be a potent agonist for LPA_5_, was a poor inducer of hypertension. The present study also suggests that LPA has other target(s) than LPA_4_ to induce transient hypertension, since LPA- and T7-induced hypertension was not suppressed completely in LPA_4_-deficient mice (Figs 3B and 6D). Although LPA activates ion channels such as TRPV1^16^, this activity does not appear to be involved in hypertension because TRP channel blockers (A784168, capsazepine and AMG9810) had no effect on the hypertensive activity of LPA (Supplementary Fig. 3). Because Y-27632 dramatically suppressed LPA-induced hypertensive response (Fig. 2), GPCR-type LPA receptors other than LPA_1–6_ that couple with Gα_12/13_ may be involved.

Use of an ATX inhibitor clearly showed that LPA is the factor in the incubated plasma that induces transient hypertension (Fig. 1D). Unlike incubated plasma which contains a high concentration of LPA (~ several µM level), the LPA level in the fresh plasma is quite low (Fig. 7B). Thus, it remained unclear if endogenous LPA in the circulation controls blood pressure. The LC-MS/MS analysis revealed that the concentration of circulating LPA in healthy mice was several tens of nM (Fig. 7B). The minimum dose of LPA to induce obvious hypertension was 0.01 mg/kg (Fig. 1B), which corresponds with about ~ 500 nM circulating LPA. Therefore, endogenous circulating LPA seems to be slightly lower than the minimum concentration to induce hypertension. Mouse plasma contains mainly unsaturated LPA, such as 18:2-LPA, which was reported to be a potent inducer of hypertension in rat^6^. In the present study, unsaturated LPA, such as 18:1-LPA and 20:4-LPA, induced hypertension more efficiently than saturated LPAs (14:0, 16:0 and 18:0) in mice (Fig. 1B). In addition, the concentration of LPA and ATX are markedly elevated in various pathological conditions. For example, unsaturated plasma LPA, such as 18:2, 20:4 and 22:6, was selectively increased in patients with acute coronary syndrome^17,18^. Upon blood coagulation, 20:4-LPA is known to be produced in activated platelets^18^. The ATX level increases in normal pregnant women in the third trimester and to a higher extent in women facing preterm delivery^19^. Therefore, it would be quite interesting to examine whether the increased unsaturated LPA affects the blood pressure in such pathological conditions.

The precise molecular mechanisms by which LPA leads to elevate blood pressure via LPA_4_ remain to be elucidated. In agreement with previous studies using rats^20^, we confirmed that pretreatment by indomethacin (an inhibitor of prostanoid synthesis) and hexamethonium (a nicotinic acetylcholine receptor antagonist that acts in autonomic ganglia) had no effect on the LPA-induced hypertensive response in mice (data not shown), showing that prostanoids and the autonomic nervous system are not involved. In this study, we showed that pretreatment with Y27632, a Rho kinase (ROCK) inhibitor, significantly suppressed LPA-induced hypertensive response (Fig. 2A). LPA_4_ is known as a Gα_12/13_-coupled GPCR according to our present data (Fig. 2B). Recent studies have shown that the Rho-ROCK pathway is a novel therapeutic target in the treatment of various cardiovascular diseases, such as pulmonary hypertension and cerebral vasospasm^21,22^. Activation of Rho downstream of G12/13 is known to elicit an actomyosin-dependent contraction of aortic smooth muscle cells in an intracellular Ca^2+^-independent manner. Thus, it is possible that an ATX/LPA/LPA_4_ axis operates upstream of the Gα_13_-Rho-ROCK pathway, possibly in aortic smooth muscle cells, and is a promising drug target.

ATX-deficient mice die around embryonic day 9.5–10.5 with profound vascular defects in both yolk sac and embryo, indicating that LPA produced by ATX has a critical role in embryonic vascular development^23^. However, none of the single LPA receptor-deficient mice showed the same phenotype, although LPA_4_-deficient mice were partially lethal due to impaired blood and lymphatic vessel formation. Thus, it remains unclear how LPA regulates embryonic vascular development. Both LPA_4_ and LPA_6_ couple mainly with Gα_12/13_ protein (Fig. 2B) to activate Rho-ROCK signaling and act to coordinately regulate cell motile activity^24^. In this study, we showed that LPA_6_-deficient mice were viable but had obvious vascular abnormalities (Fig. 5), which is partially similar to the phenotype of LPA_4_-deficient mice. In contrast, we were unable to produce *Lpar4*/*Lpar6*-double null mice. It is also notable that the number of offspring carrying a single wild-type *Lpar4* allele on an *Lpar6*-deficient background was less than expected (Table 1). These data raise the possibility that both LPA_4_ and LPA_6_ generate angiogenic signaling, which is essential for embryonic vascular development and is missing in ATX-deficient mice. Interestingly only LPA_6_-deficient mice exhibited impaired pressor response to various vasopressors (Fig. 4). Therefore, further studies are necessary to investigate the precise and differential roles of LPA_4_ and LPA_6_ signaling in embryonic vascular development.

In summary, we propose a novel mechanism of LPA-induced transient hypertension in which unsaturated LPA produced by ATX stimulates mainly LPA_4_ and induces the hypertensive response through Gα_12/13_-Rho-ROCK signaling.

## Methods

### Reagents

LPA (1-myristoyl (14:0), 1-palmitoyl (16:0), 1-stearoyl (18:0), 1-oleoyl (18:1), 1-arachidonyl (20:4)) was purchased from Avanti Polar Lipids. LPA and T-series compounds were dissolved in PBS containing 0.1% fatty acid free BSA (Sigma) and stocked at −20 °C. Biotinylated *Griffonea Simplicifolia* I isolectin B4 was purchased from Vector Laboratories. PTX and Y-27632 were from Calbiochem and Wako, respectively. The ATX inhibitor (ONO-8430506)^4^ was kindly donated by ONO Pharmaceutical Company.

### Mouse breeding

Mice (C57BL6 and ICR, male, 8 weeks) were purchased from SLC Japan. LPA_1_, LPA_2_, LPA_3_ and LPA_4_ knockout (KO) mice were established as described previously^14,25,26^. LPA_6_ KO mice with a mixed 129/Sv and C57BL/6 were obtained from Deltagen (San Carlos, CA). Mice were housed under specific pathogen-free conditions in an air-conditioned room and fed standard laboratory chow ad libitum. All mice were treated in accordance with the protocol approved by the Animal Ethics Committee of the Graduate School of Pharmaceutical Sciences, Tohoku University, Japan.

### Whole-mount staining and immunofluorescence staining

Immunostaining of flat-mount retinas was performed according to a previously described method^27^.

### Measurement of blood pressure in mice

Male mice anesthetized with urethane (1.5 mg/kg, *i*.*p*.) were placed on a heating plate at 40 °C. Under a stereoscopic microscope, the trachea was exposed and cannulated. Subsequently, a polyethylene-tipped cannula (PE-60 tubing) was inserted into the left carotid artery to monitor arterial pressure. The arterial cannula was connected to a transducer and blood pressure signals were recorded using PowerLab4/25 (Bio Research Center, Nagoya, Japan). To analyze acute blood pressure response, a second catheter was placed in the right femoral vein to infuse agonists. Mice received a bolus injection (100 µl/time) at 5–10 min intervals. For pharmacological studies, PTX (30 µg/kg, *i*.*v*.) was dissolved in PBS and administered 24 hr and 48 hr before injection of LPA. Mice were treated with saline dilutions of Y-27632 (0.1–10 mg/kg, *i*.*v*.) 5 min before injection of LPA.

### LC-MS/MS analysis

Lipids were extracted from plasma using methanol (including 17:0-LPA as internal standard; final concentration was 100 nM) as described previously^28^ and stored at −80 °C. LC-MS/MS analysis was performed according to a previously described method with minor modifications^28^. In this study, we used an LC-MS/MS system that included an Ultimate3000 HPLC and TSQ Quantiva triple quadropole mass spectrometer (Thermo Fisher Scientific). LPA analyses were performed in the multiple reactive monitoring (MRM) in negative mode^28^. LC was performed using a reverse phase column (CAPCELL PAK C18 (1.5 mm I.D. x 250 mm, particle size was 3 µm)) with a gradient elution of solvent A (5 mM ammonium formate in 95% (v/v) water, pH 4.0) and solvent B (5 mM ammonium formate in 95% (v/v) acetonitrile, pH 4.0) at 200 µL/min. Gradient conditions were as follows: hold 50% B for 0.2 min, followed by a linear gradient to 100% B over 11.8 min, hold 100% B for 5 min, return to the initial condition over 0.5 min, and maintain for 2.5 min until the end of run (total run time 20 min).

### AP-TGFα shedding assay

This assay was conducted according to a previously described method with several modifications^13^. To improve signal detection, HEK293 cells were transfected with Gα chimeric proteins and treated with the LPA_1–3_ antagonist, Ki16425. The siRNAs were transfected into cells by using Lipofectamine RNAiMAX. We validated siRNA mediated knockdown of Gα_12_ and Gα_13,_ previously^13^. Two days post-transfection, cells were co-transfected with mouse FLAG-LPA_1–6_, AP-TGFα and Gα chimera by using Lipofectamine2000. After 24 hr, cells were resuspended in HBSS buffer, seeded in 96 well assay plates and stimulated with ligands and 10 µM Ki16425 for 1 hr. 80 µl of conditioned media were transferred to new 96 well plates and mixed with an equal volume of *p*-NPP solution. AP activity was calculated by the measurement of absorbance at 405 nm with a microplate reader (Molecular Devices).

### Calculations

Agonist activities in the reporter gene and shedding assays were estimated as described previously^29^. The EC_50_ value and E_max_ values were calculated by fitting a logistic equation to the data by nonlinear regression analysis. The RIA (relative intrinsic activity) values, which indicate the relative potency of an agonist to LPA, were determined.

### Statistical analysis

Unpaired Student’s t-test, one-way ANOVA followed by Tukey’s post hoc test and multiple comparisons t-test were used for the statistical analysis. A value of *P* < 0.05 was considered statistically significant.

## Supplementary information

Supplementary Figure 1–3
