# Supplementary material for: Molecular mechanism of lysophosphatidic acid-induced hypertensive response
Source: Sci Rep. 2019 Feb 25;9:2662. doi: 10.1038/s41598-019-39041-4 (PMC6389983; doi:10.1038/s41598-019-39041-4)
Supplement: Supplementary file 1 — Supplementary Figure 1–3 [file 41598_2019_39041_MOESM1_ESM.pdf]

Supplementary Information
(Supplementary Figure 1-3)

Molecular mechanism of lysophosphatidic acid-induced hypertensive response

Kuniyuki Kano[1,2,3], Hirotaka Matsumoto[1], Asuka Inoue[1,3,4], Hiroshi Yukiura[1], Motomu Kanai[5], Jerold Chun[6], Satoshi Ishii[7], Takao Shimizu[8,9] and Junken Aoki[1,2,3]

[1] Laboratory of Molecular and Cellular Biochemistry, Graduate School of Pharmaceutical Sciences, Tohoku University, [2]AMED-CREST, [3]LEAP, [4]PRIME, Japan Science and Technology Corporation, [5]Synthetic Organic Chemistry Laboratory, Graduate School of Medicine, University of Tokyo, [6]Department of Molecular and Cellular Neuroscience, Dorris Neuroscience Center, The Scripps Research Institute, [7]Department of Immunology, Akita University Graduate School of Medicine, [8]Department of Lipid Signaling, National Center for Global Health and Medicine, [9]Lipidomics, Graduate School of Medicine, University of Tokyo

Address all correspondence to:

Junken Aoki:	or	Kuniyuki Kano
E-mail : jaoki@m.tohoku.ac.jp		E-mail : k-kano@m.tohoku.ac.jp
Telephone +81-22-795-6860		Telephone +81-22-795-6862
FAX +81-22-795-6859		FAX +81-22-795-6859

Supplementary Figure legends

Supplementary Figure 1. LPA induced-hypertension in $LPA_{1\sim3}$ KO mice.
Original recording of mice blood pressure. LPA (1.4 mg/kg) was intravenously injected into mice.

Supplementary Figure 2. LPA_4 and LPA_6 single KO mice showed normal blood pressure and heart rate.
Under urethane anesthesia, blood pressure and heart rate were monitored in LPA_4 KO mice (A) and LPA_6 KO mice (B). Data represented as mean ± S.E.

supplementary Figure 3. TRP channel blocker had no effect on the LPA-induced hypertension.
Original recording of mice blood pressure. A mixture of TRP channel blocker (A784168 (0.1 mg/kg), Capsazepine (2.5 mg/kg) and AMG9810 (0.015 mg/kg)) was intravenously administered followed by LPA (1.4 mg/kg) into wild-type mice. Arrow indicates the time point of injection of LPA.

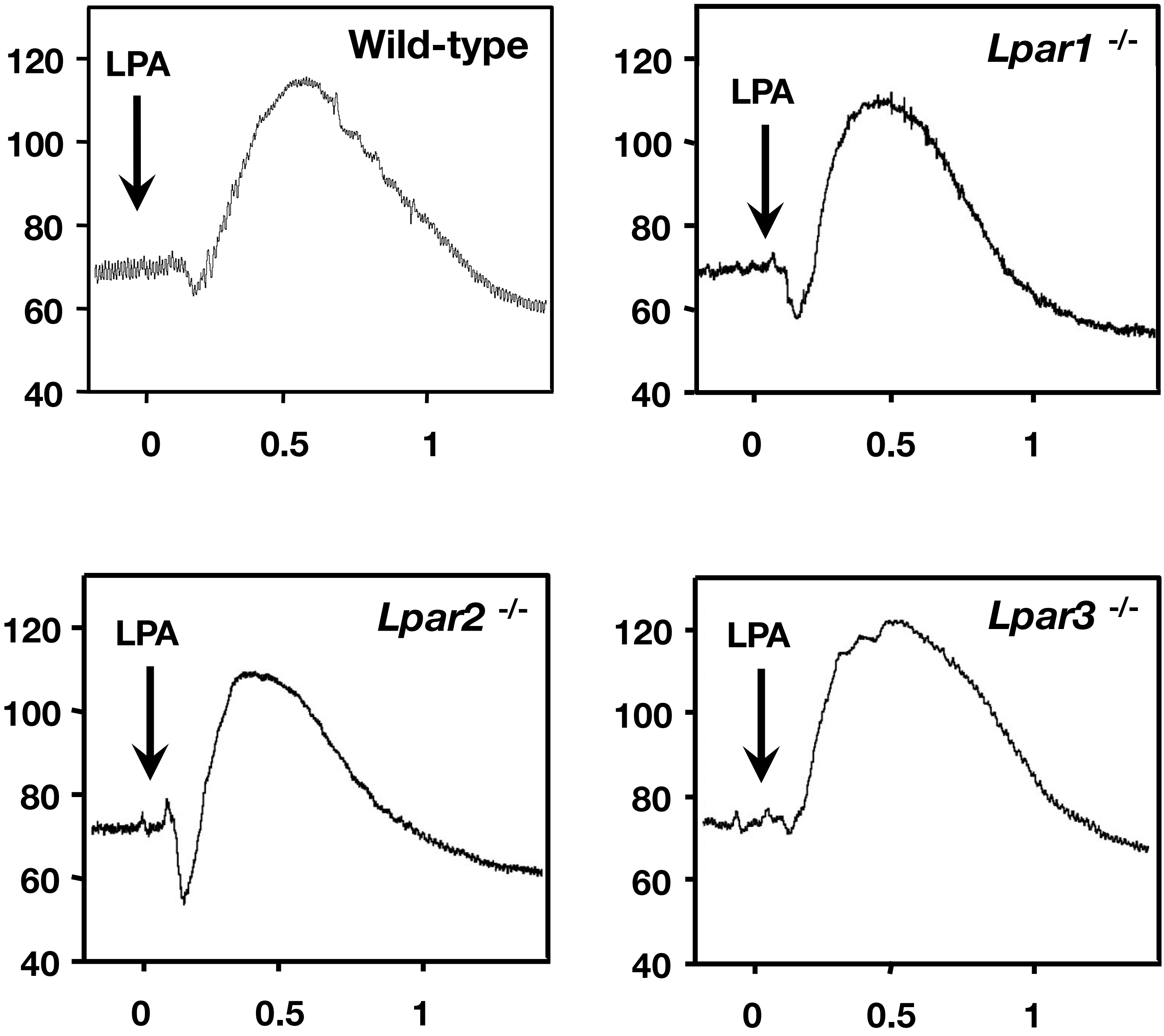

Supplementary Figure 1.
LPA induced-hypertension in $LPA_{1\sim3}$ KO mice

(A)

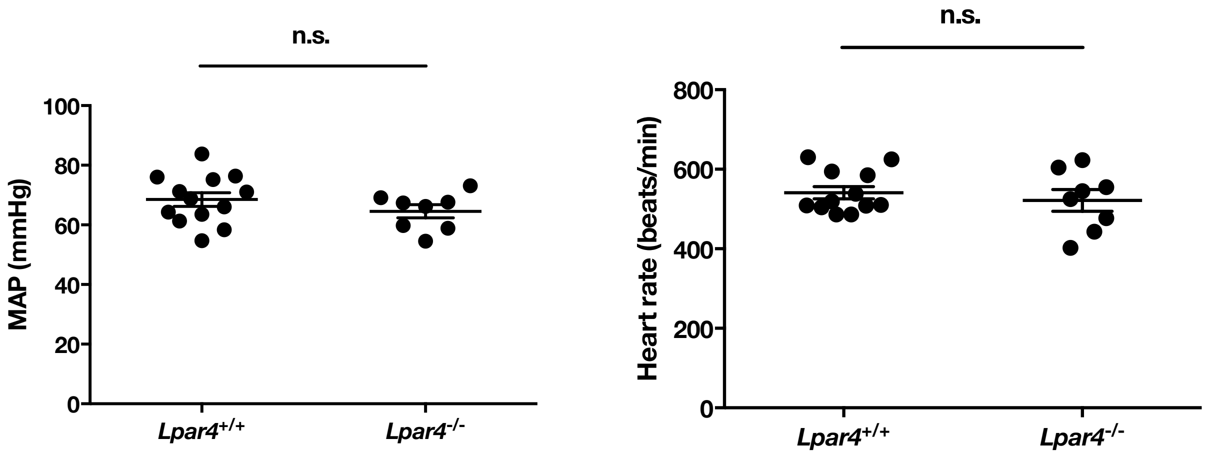

(B)

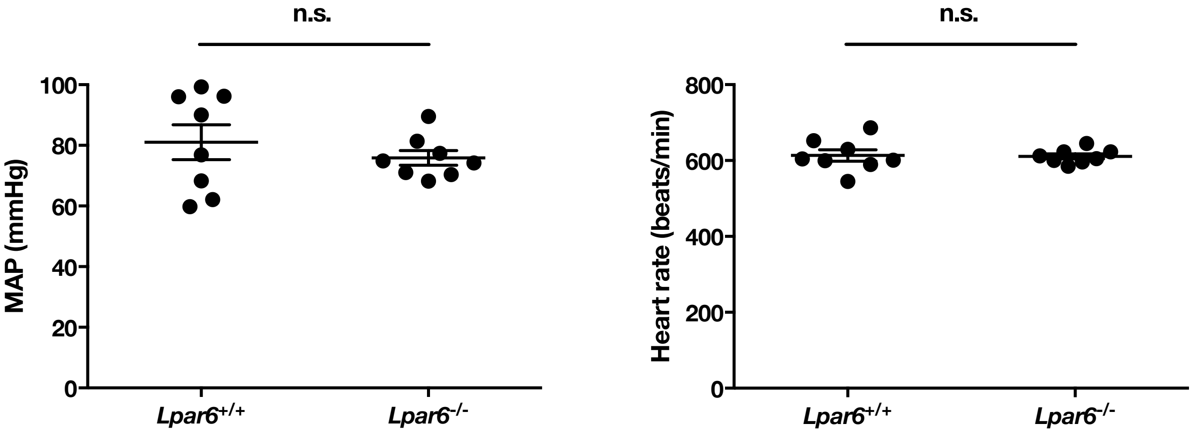

Supplementary Figure 2.
LPA_4 and LPA_6 single KO mice showed normal blood pressure and heart rate

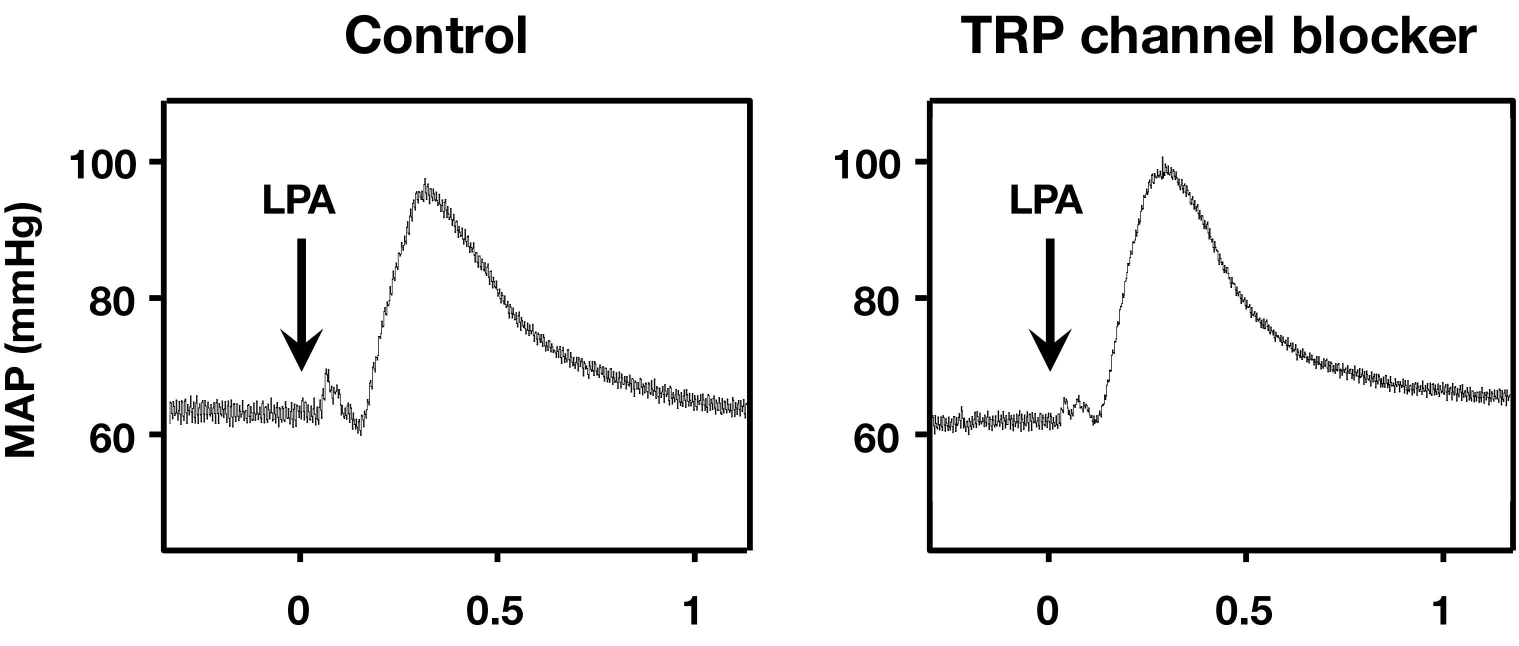

Supplementary Figure 3.
TRP channel blocker did not affect LPA-induced hypertension
